# Variable-rate in corn sowing for maximizing grain yield

**DOI:** 10.1038/s41598-021-92238-4

**Published:** 2021-06-16

**Authors:** Eder Eujácio da Silva, Fábio Henrique Rojo Baio, Daniel Fernando Kolling, Renato Schneider Júnior, Alex Rogers Aguiar Zanin, Danilo Carvalho Neves, João Vítor Pereira Ferreira Fontoura, Paulo Eduardo Teodoro

**Affiliations:** Universidade Federal de Mato Grosso do Sul, Chapadão do Sul, MS Brazil

**Keywords:** Plant sciences, Environmental sciences

## Abstract

Sowing density is one of the most influential factors affecting corn yield. Here, we tested the hypothesis that, according to soil attributes, maximum corn productivity can be attained by varying the seed population. Specifically, our objectives were to identify the soil attributes that affect grain yield, in order to generate a model to define the optimum sowing rate as a function of the attributes identified, and determine which vegetative growth indices can be used to predict yield most accurately. The experiment was conducted in Chapadão do Céu-GO in 2018 and 2019 at two different locations. Corn was sown as the second crop after the soybean harvest. The hybrids used were AG 8700 PRO3 and FS 401 PW, which have similar characteristics and an average 135-day cropping cycle. Tested sowing rates were 50, 55, 60, and 65 thousand seeds ha^−1^. Soil attributes evaluated included pH, calcium, magnesium, phosphorus, potassium, organic matter, clay content, cation exchange capacity, and base saturation. Additionally, we measured the correlation between the different vegetative growth indices and yield. Linear correlations were obtained through Pearson’s correlation network, followed by path analysis for the selection of cause and effect variables, which formed the decision trees to estimate yield and seeding density. Magnesium and apparent electrical conductivity (EC_a_) were the most important soil attributes for determining sowing density. Thus, the plant population should be 56,000 plants ha^−1^ to attain maximum yield at EC_a_ values > 7.44 mS m^−1^. In addition, the plant population should be 64,800 plants ha^−1^ at values < 7.44 mS m^−1^ when magnesium levels are greater than 0.13 g kg^−1^, and 57,210 plants ha^−1^ when magnesium content is lower. Trial validation showed that the decision tree effectively predicted optimum plant population under the local experimental conditions, where yield did not significantly differ among populations.

## Introduction

Sowing density for corn (*Zea mays* L.) cultivation is of paramount importance to obtain high grain yields^1,2^. Specifically, the average sowing density recommended for current corn hybrids grown in Brazil ranges from 45,000 to 65,000 plants ha^−1^^3,4^, depending on the hybrid and the sowing date. In general, yield per unit area increases with increasing plant population density, while plant yield decreases, owing to the stress caused by plant overcrowding^1,3^.

Several studies have examined maize population density under varying soil conditions, locations, and, especially, sowing seasons^4–6^ or the second sowing season after soybean harvest. These studies are particularly relevant in cases where conditions of low precipitation prevail during maize cropping in the second season, which comprises the late rainy and the early dry seasons under tropical climate features^7^, including rainy summers and dry winters. These climate characteristics offer a limited amount of precipitation during the second cropping season.

The main factors that determine the optimum sowing density are soil moisture^8,9^, soil nutrient availability^10^, and choice of hybrid^1,3^. Soil water content correlates highly with apparent electrical conductivity (EC_a_) of the soil^9,11^, whose relatively simple measurement is a low-cost, convenient tool for characterizing soil physicochemical properties^12^, such as moisture, clay content, and organic matter. The measurement of these variables is important to attain maximum crop yield, as they provide a convenient means for estimating the soil potential to nourish a healthy and vigorous plant development with adequate water and nutrient supply^13^.

In precision agriculture, proper mapping of soil attributes—other than EC_a_—may assist decision-making aimed to recommend crop input levels. Plant emergence conditions usually influence population variation, quality of land preparation and sowing operations, and seed vigor. To date, there have been no studies focused on researching variable-rate-seeding (VRS) performed in the same area. This technology requires preparing a prescription map that points out how the electronic seeder controller should change seed spacing within rows to achieve a variable seeding rate as convenient^14^. Indeed, VRS can be performed by several commercially available seeders equipped with electronic controllers for seed-dosing discs that can vary the seeding rate by changing the spacing between seeds along the row^14^. Nonetheless, even with the available instruments, the parameters for defining the appropriate corn sowing rates to be applied remain subjective. Therefore, the hypothesis tested in this study was that it should be possible to vary the corn seed population according to soil attributes to maximize grain yield. Consistently, the objectives of this study were to identify the soil attributes that affect grain yield and generate a model to recommend sowing rates as a function of these attributes.

## Material and methods

### Experimental sites

**Figure 1:**
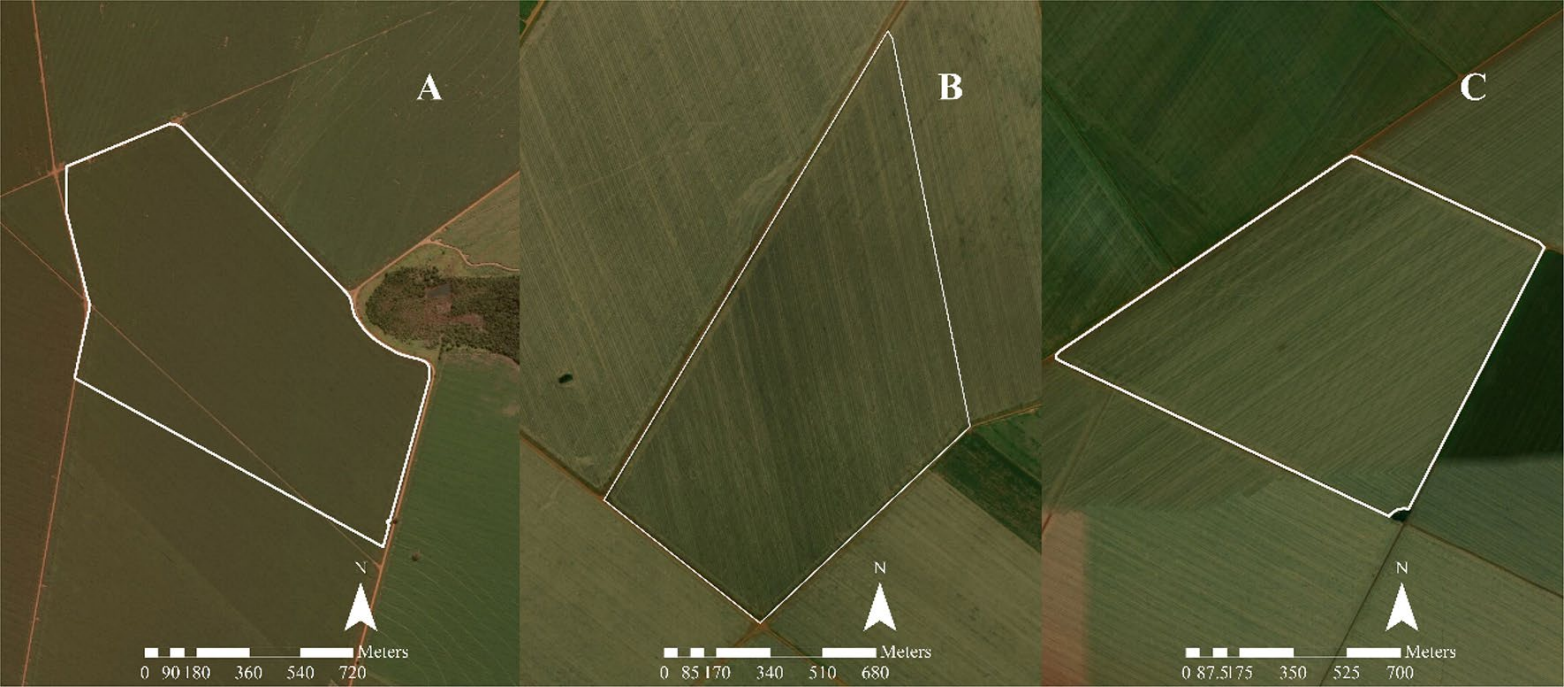
Experimental sites in the Alto Formoso (**A**) and Porto Seguro (**B**, **C**) farms. The software used to create this Figure was ArcGIS Desktop (v10.5, https://desktop.arcgis.com/en/system-requirements/10.5/).

The experiment was conducted at four field locations within Chapadão do Céu/GO/Brazil in the 2017/2018 and 2018/2019 cropping seasons. Site A was located in the Alto Formoso farm (near 18°21ʹ43.11″S, 52°41ʹ19.01″W), with an average elevation of 810 m. Sites B, C, and D were located in the Porto Seguro farm (near 18°21ʹ26.00″S, 52°34ʹ10.71″W), with an average elevation of 820 m (Fig. 1). Sites B and C were the same but in two different crop seasons. Site D was used to compare the results with those of the fixed stand. The soil at the sites is classified as dystrophic red latosol^15^, with a texture ranging from 180 to 822 g kg^−1^ of clay.

### Experimental design

**Figure 2:**
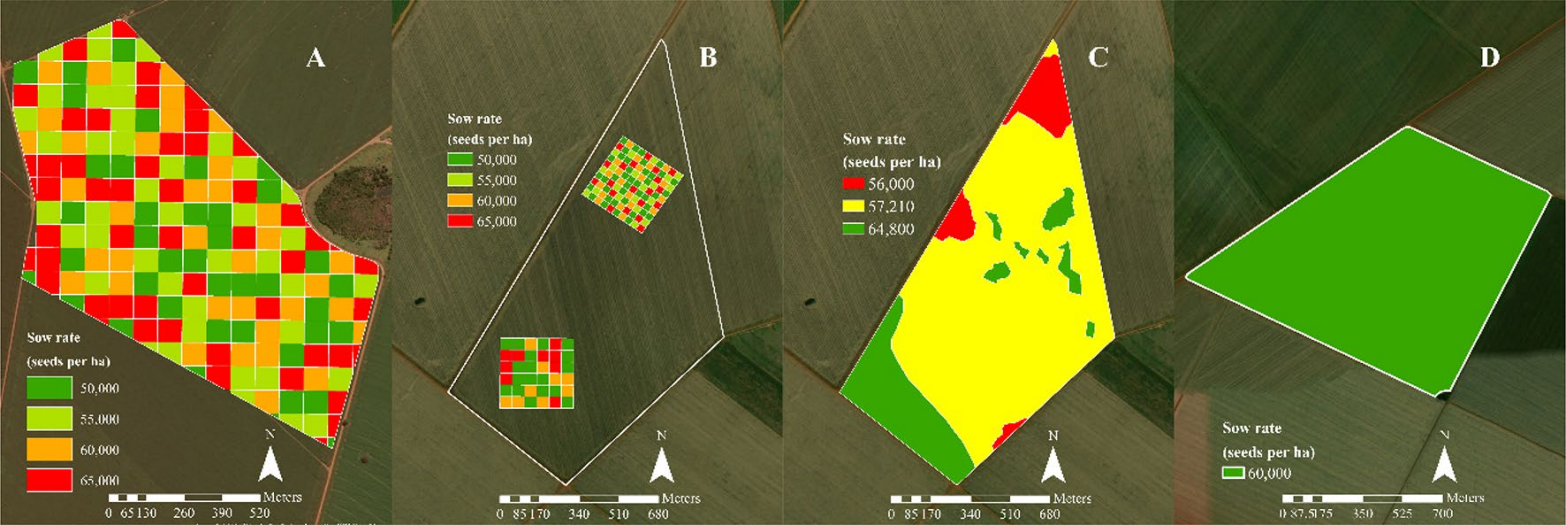
Experimental plots and planned seeding rates for sites **A** and **B** in the 2017/2018 cropping season and sites **C** and **D** in the 2018/2019 cropping season (used to validate the results). The software used to create this Figure was ArcGIS Desktop (v10.5, https://desktop.arcgis.com/en/system-requirements/10.5/).

The experiment was laid in a completely randomized design with four seed populations (50,000, 55,000, 60,000, and 65,000 seeds ha^−1^) randomly distributed in 136 plots at each site (sites A and B) in 2018. At site A, the 136 plots were sized to fill the 87 ha field entirely, measuring 80 × 80 m (Fig. 2A), disregarding field boundaries. Thus, the plots were divided into two sets of different dimensions at this site: site 2a contained 36 plots of 50 × 50 m, and site 2b contained 100 plots of 30 × 30 m (Fig. 2B). These dimensions were different to provide a more significant variability in the results as a function of plot size. Prescription maps containing sowing rates were processed using the Geographic Information System (GIS) software ArcGIS 10.5 (ESRI, Redlands, CA, USA).

The experiment was validated in the 2019 crop season at site C (Fig. 2C) in a total area of 103 ha. Variable-rate-seeding (VRS) was performed based on the results obtained from the plots of the 2018 agricultural year. Site D (94.5 ha, Fig. 2D) was sown under a conventional system (CVS) to compare the results with those obtained from the VRS system applied at site 2.

### Climate and weather conditions

**Figure 3:**
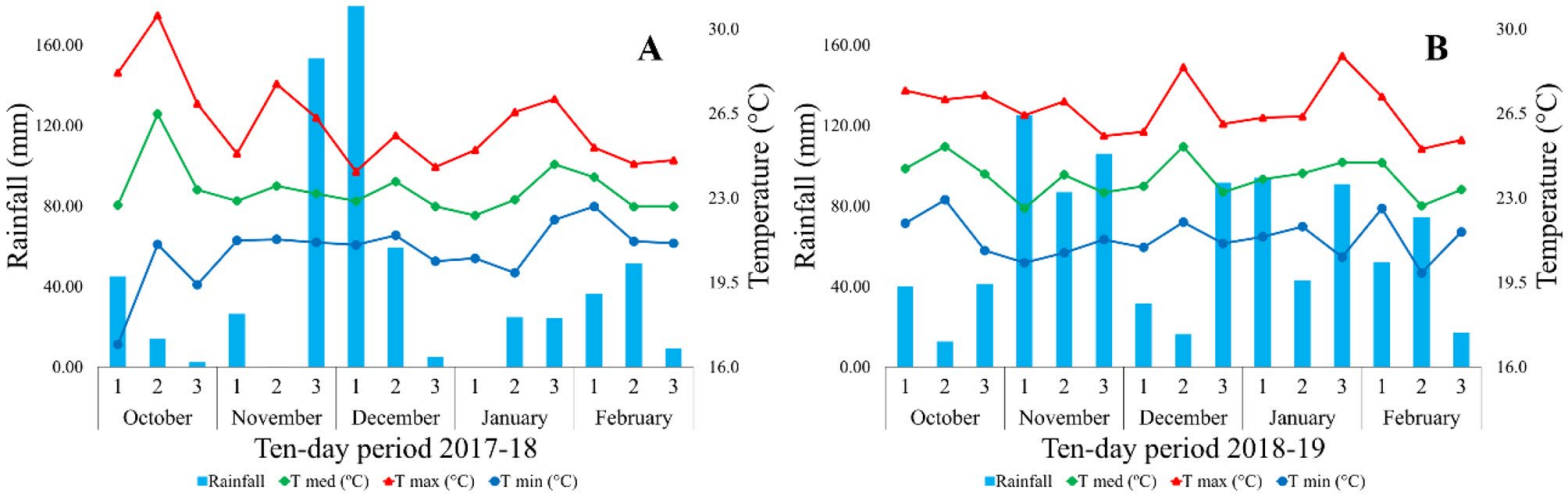
Mean rainfall (mm), maximum, minimum, and mean temperature (°C) recorded at 10-day intervals during the corn cultivation in 2018 (**A**) and 2019 (**B**). Chapadão do Céu. The software used to create this figure was Microsoft Excel (v1804, https://www.microsoft.com).

The trial was conducted during the 2018 and 2019 agricultural years. The climate of the region is characterized as tropical with a dry winter season (Aw) according to the Köppen climate classification. Weather data were obtained through an automatic weather station installed at Porto Seguro farm and compiled through the ZeusAgro platform (Zeus Agrotech, Uberlândia, Brazil). Data were pooled at 10-day intervals during the experimental period. In 2018, the average 10-day precipitation ranged between 0 and 193 mm, totaling 657.8 mm during the period when the crop was in the field. The ambient temperature fluctuated between 8.3 and 32.2 °C. In 2019, the average 10-day precipitation ranged between 0 and 100 mm, totaling 581.1 mm during the experimental period, and ambient temperature ranged from 7.9 to 32.7 °C (Fig. 3).

### Soil properties

Soil chemical and physical attributes were measured before installing the experimental field by regular grid sampling of 100 m sideways, at a depth of 0.0–0.2 m in the sampling areas (Table 1). Then, 10% of the random sampling points in each area were added to this sample grid to analyze the spatial variability at a shorter distance. A total of 100, 103, and 95 sampling points were included for areas 1, 2, and 3, respectively.

**Table 1:** Means of soil attributes in the experimental areas obtained from a total of 300 sampling points.

Area	pH	Soil attributes								
		Ca	Mg	K	P	CEC	V %	OM	Clay	EC_a_
		g kg^-1^				cmolc kg^-1^	%		G kg^−1^	mS m^−1^
1	5.17	0.679	0.125	0.089	0.009	7.98	57.9	3.0	646.9	9.41
2	5.24	0.772	0.108	0.060	0.019	8.44	57.6	3.2	436.98	6.44
3	5.11	0.882	0.143	0.071	0.013	10.74	53.0	2.8	547.0	9.42

Ca, calcium; Mg, magnesium; CEC, cation exchange capacity; K, potassium; P, phosphorus; V%, base saturation; OM, organic matter; Clay, clay content; EC_a_, soil apparent electrical conductivity.

The EC_a_ attribute was measured using the equipment Veris Q1000. This sensor has four electrodes connected to flat metal discs and are arranged side by side, making contact with the ground to a depth of 0.05–0.07 m^16^. An electronic unit controls the emission and reception of disc loads for measuring EC_a_, where the measured value is linked to the coordinates from the global navigation satellite system (GNSS) to georeference the collected data, which were stored in a flash memory device connected to a control panel. This implementation was coupled to a Honda (Sumaré, Brazil) all-terrain vehicle (ATV) that provided motor and electric power to the system. The data readings were taken on parallel swathing arranged every 20 m.

The maps of all variables were prepared using the ordinary Kriging interpolation method^17,18^. Geostatistical analysis was performed using the ArcGIS 10.5 software, followed by the precepts of spatial continuity^18^. The modeled semivariograms were determined based on the lowest mean standard error calculated by cross-validation^18^ using the same software.

### Establishment of the experiment

The plots were established in the areas defined for the second crop after soybean harvesting. Site A was sown on February 11, 2018, with a John Deere DB planter (John Deere, Horizontina, Brazil) with a row spacing of 0.45 m. The seeds used were of corn hybrid AG 8700 PRO3 (Agroceres Company). Site B was sown on February 8 and 10, 2018, using a John Deere 2117 planter with the same row spacing as site A. The planter was equipped with a V-Drive seed distribution system (Precision Planting, Tremont, IL, USA). The seeds used were of corn hybrid 2A401 PW (Forseed Company). Both hybrids showed an average cropping cycle of 135 days and similar phenological characteristics. Plant materials used in this study complied with relevant institutional, national, and international guidelines and legislation.

Sites C and D (experimental control treatment with the conventional seed system, CVS, at a fixed seeding rate) were sown in the second annual crop cycle, after soybean harvest, between February 10 and 11, 2019, with the John Deere 2117 planter at a row spacing of 0.45 m. The corn hybrid was the same (FS 401 PW) as that used in the VRS plots. The sowing of site D was conventionally performed at a fixed rate of 60,000 seeds ha^−1^.

### Fertilization and plant health treatment

Soil amendment using lime (1.5 ton ha^−1^) and gypsum (300 kg ha^−1^) was performed before sowing the first crop to increase soil pH and improve base saturation conditions. Fertilization at sowing was performed according to soil analysis results, following the recommendations for the region^19^. Sowing fertilization was carried out in area 1 using a self-propelled solids distributor Hercules 5.0, (Stara, Não me Toque, Brazil) and in the sowing furrow in area 2 by the planter. Top-dressing fertilization was carried out in the two areas using 100 kg N ha^−1^ (150 kg urea ha^−1^) at the V4 phenological stage of corn.

### Assessment of planting

Planting was evaluated in the three plot groups 39 days after emergence (DAE), when the corn crop was at the V6 phenological stage, by counting the number of plants along 10 m in three sowing rows. The sum of the three-line counts served to generate an average used to verify the planned populations.

The population of each cluster was grouped into classes. Verification was necessary to determine the actual standing plant populations during the experimental period, owing to the germination, survival, and purity indexes of the hybrid seeds used.

### Grain yield assessment

Harvesting was performed using three harvester models. In area 1, two different harvesters were used: S680 (John Deere) and CR8.90 (New Holland, Sorocaba, Brazil). In areas 2 and 3, harvesting was performed by model 9230 (Case, Piracicaba, Brazil) and model S680 (John Deere). The variability in area productivity was determined using the FieldView Cab harvest monitoring system (Climate Field View, São Paulo, Brazil), which allows data from the different harvesting systems of each harvester to be combined. The harvest monitoring systems installed on the machines were calibrated before harvesting by an electronic system weighing cell load.

The raw data for grain yield were filtered based on the variance, and the upper and lower cutoff limits were determined according to the methodology suggested by Tukey^20^. After interpolation using the ordinary Kriging methodology^17,21^, point productivity information correlated with the other components was obtained by averaging the points measured within 10 m of the control sampling point to reduce point variability.

### Statistical analysis

Because of the large number of variables in each class, a correlation network was used to graphically express the results, where the proximity between nodes (traces) was proportional to the absolute value of the correlation between these nodes^22^. The determination of the most relevant variables in terms of grain yield was achieved by a trial analysis using the Genes program^23^. The variables selected as most significant were used to generate a decision tree algorithm, considering grain yield as the dependent variable. In this process, 80% of the data were used for algorithm training and 20% for validation. Model accuracy was assessed by the correlation between estimated and observed values at each stage. This analysis was performed using the Genes software.

The analysis of variance (ANOVA) was performed to compare grain yield among the recommended VRS rates by decision tree (Area C) with grain yield under CVS (Area D). The relative deviation coefficient (RDC) expresses the dissimilarity between two maps in the module^24^. The calculation was performed using Eq. (1). All variable values were converted to percentage values to compare the different units. The grain yield map was considered as the reference (standard) for comparison with the other maps.

1 $$RDC={\sum }_{i=1}^{n}\left|\frac{{P}_{ij}-{P}_{iref}}{{P}_{iref}}\right|*\frac{100}{n}$$

where $n$ is the number of points, ${P}_{ij}$ is the value of the variable in each specific sampling point, and ${P}_{iref}$ is the value of the reference variable for the same sampling point.

## Results and discussion

### Planting

**Figure 4:**
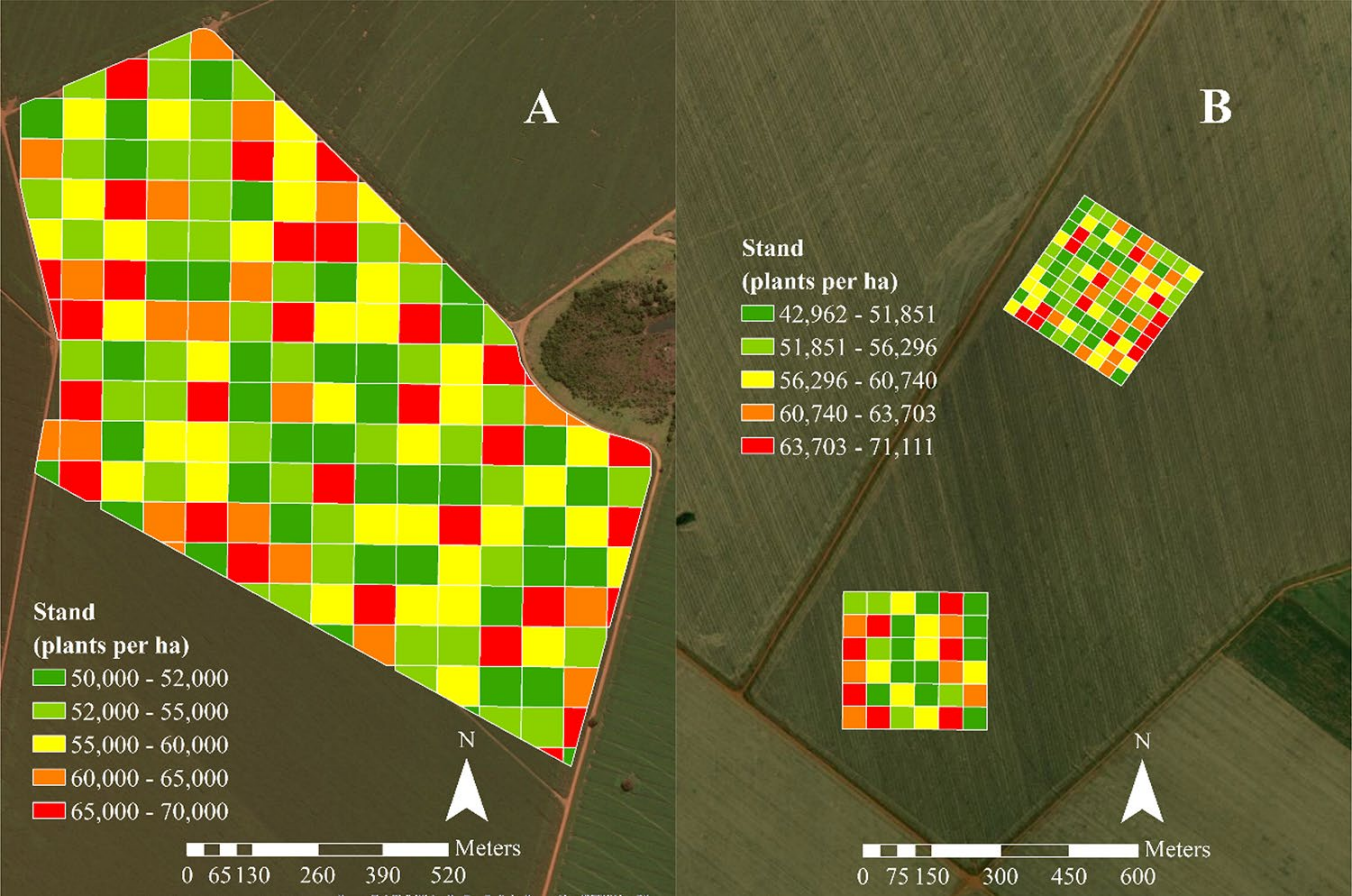
Final plant stands in sites **A** and **B** used for path analysis and tree decision and in area C used to validate results. The software used to create this Figure was ArcGIS Desktop (v10.5, https://desktop.arcgis.com/en/system-requirements/10.5/).

The final plant stands varied according to the planned population. This variation was up to ± 14% of the initial treatments, between 42,962 and 71,111 plants ha^−1^ (Fig. 4). According to our seed suppliers, the recommended population for the dates when sowing was performed was 55,000 plants ha^−1^. However, sowing density variations of − 10%, − 10%, and − 20% of the recommended population occurred. The − 20% variation was not realized, as a population of 45,000 plants ha^−1^ was not recommended for these hybrids, as the potential weed infestation at such a low planting density may be difficult to control because corn plants do not offer sufficient ground cover, thus allowing weed growth. The final mean actual population was 57,036 plants ha^−1^.

### Relationship between VSR and soil attributes

**Figure 5:**
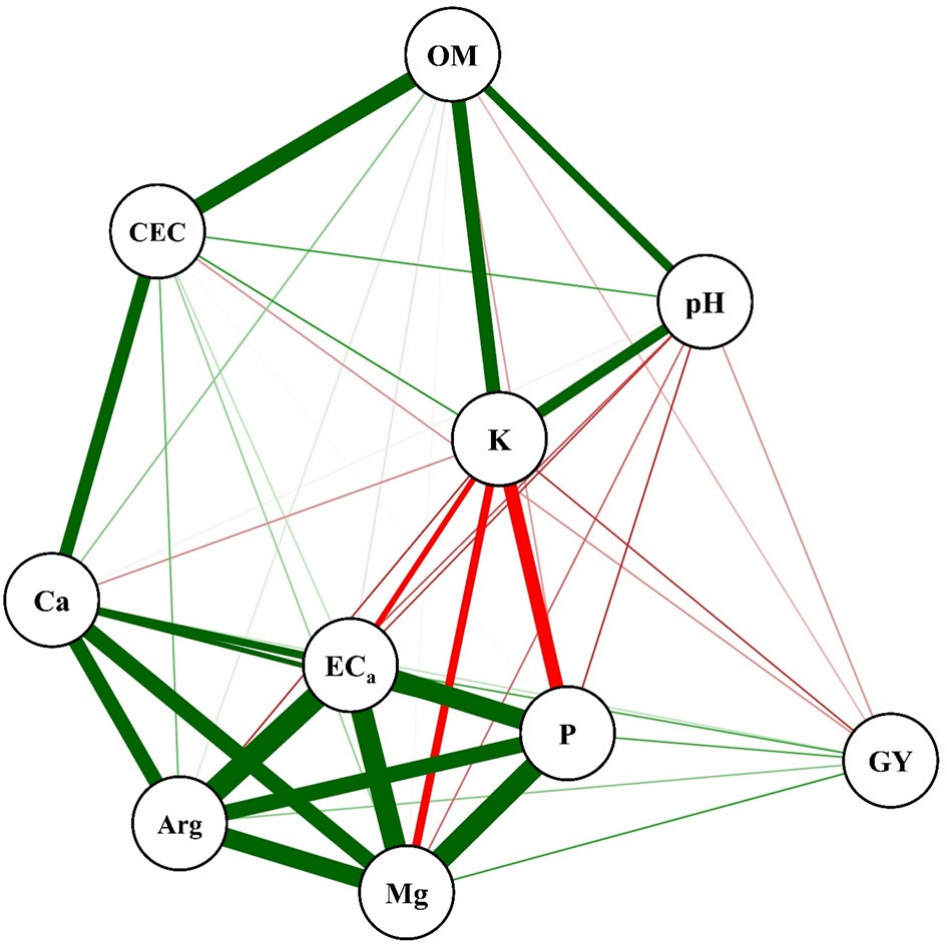
Pearson correlation network between soil attributes pH, calcium (Ca), magnesium (Mg), cation exchange capacity (CEC), potassium (K), phosphorus (P), organic matter (OM), clay, apparent electrical conductivity (EC_a_), and grain yield (GY). The package used of R to create this figure was qgraph (v1.6.9, https://cran.r-project.org/web/packages/qgraph/index.html).

Figure 5 shows the network of correlations between soil attributes measured in areas 1 and 2. The EC_a_, Mg, clay, phosphorus (P), and calcium (Ca) were positively correlated with each other and with grain yield (GY). Cation exchange capacity (CEC), potassium (K), organic matter (OM), and pH were positively correlated with each other; however, their correlation with GY was negative and inversely proportional.

Under the conditions found in the experimental sites, which cannot be extrapolated to any other area, as these are local conditions associated with a specific management scheme, K levels are directly affected by soil acidity corrected with lime, as the increase in Ca and Mg contents can inhibit K absorption by the roots, thus promoting a K deficiency; additionally, competitive inhibition occurs by membrane exchange sites and root cells, where the antagonistic effect is most evident between Mg and K^25,26^. In addition, the application of agricultural gypsum may favor K leaching, thereby reducing available K levels. Another factor that influences the availability of K for second-crop corn is the high soil nutrient-mining by soybean plants^27^.

**Figure 6:**
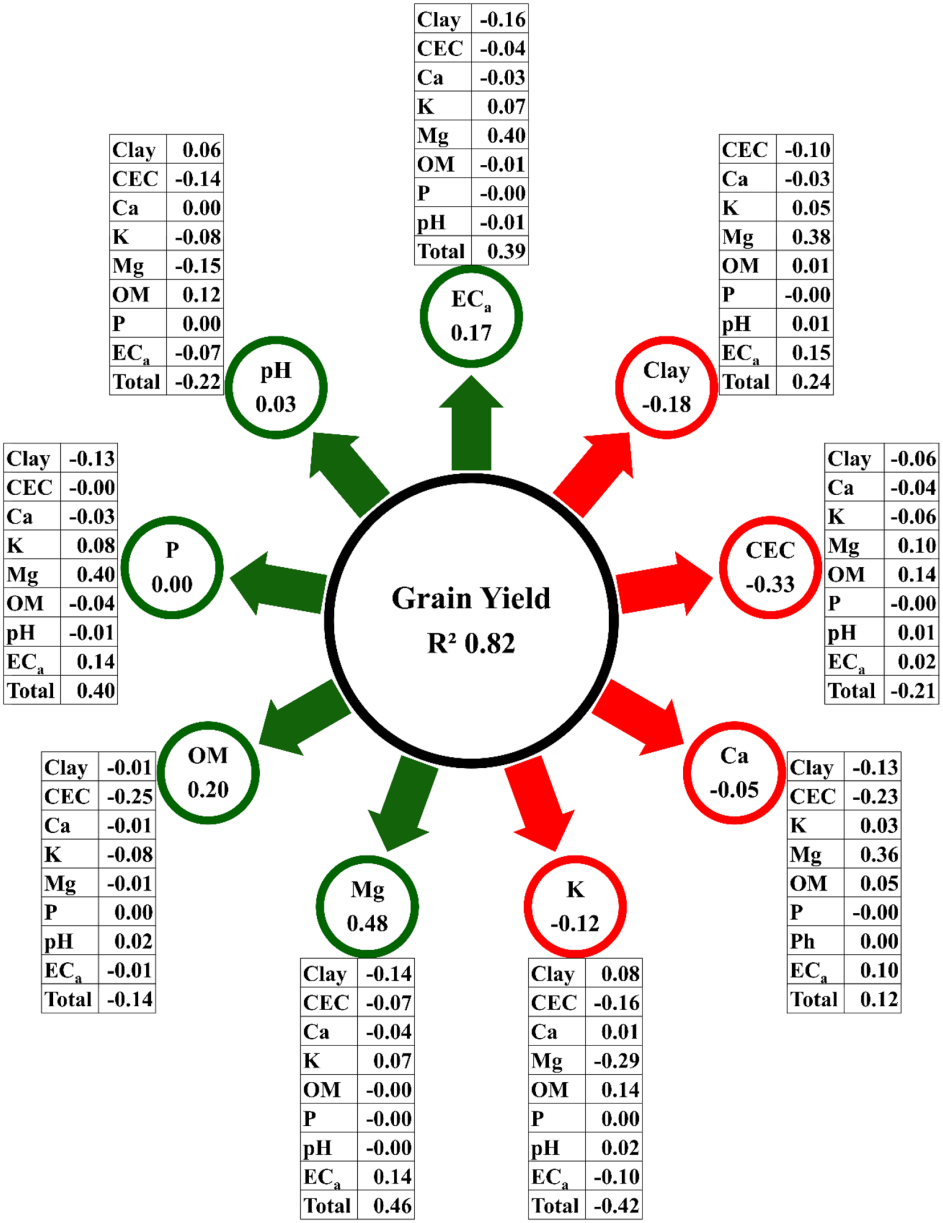
Path analysis of soil variables as a function of grain yield. Softwares used to create this figure were Genes (v1999.2019.44, http://arquivo.ufv.br/dbg/genes/gdown1.htm) and Corel draw (vX7, https://www.corel.com/).

Although important, Pearson’s correlation coefficient may produce misconceptions about the relationship between two variables and may not be a true measure of a putative cause-effect relationship. A high or low correlation coefficient between two variables may result from the effect of a third variable or group of variables on the pair, not revealing the exact relative importance of the direct and indirect effects of these factors^28^. Therefore, a path analysis was performed (Fig. 6) to investigate the cause and effect relationships detected. This analysis provides detailed knowledge of the variables' influence and justifies the existence of high- and low-magnitude positive and negative correlations between the studied variables^29^.

The results obtained in the trail analysis showed that the factors that best explained GY were Mg and EC_a_, with a direct effect of 0.48 and 0.17 and an indirect effect of 0.46 and 0.39, respectively. Mg, the central element in the chlorophyll molecule, was the most prominent nutrient in the trial analysis^26^; thus, the crop showed a higher GY where nutrient availability was greater. This result suggests that Mg was the most important nutrient for yield estimation, as GY was also higher when Mg levels were higher. Even with adequate Mg content for grain production, according to soil analysis results^19^, the lack of Mg limited the maximum development of corn plants. Therefore, Mg was the nutrient that most strongly influenced the productivity results.

In turn, EC_a_ is highly normally correlated with soil water content^8,9^ and, together with clay content, EC_a_ assists soil type classification^13^. The EC_a_ results obtained in this study suggest that, in order to obtain high GY in second-crop corn, it is necessary for any water deficit occurring during the crop cycle to be as small as possible; additionally, soils protection with mulch, clay content at the average level, and abundant rainfall, all guarantee adequate crop development.

**Figure 7:**
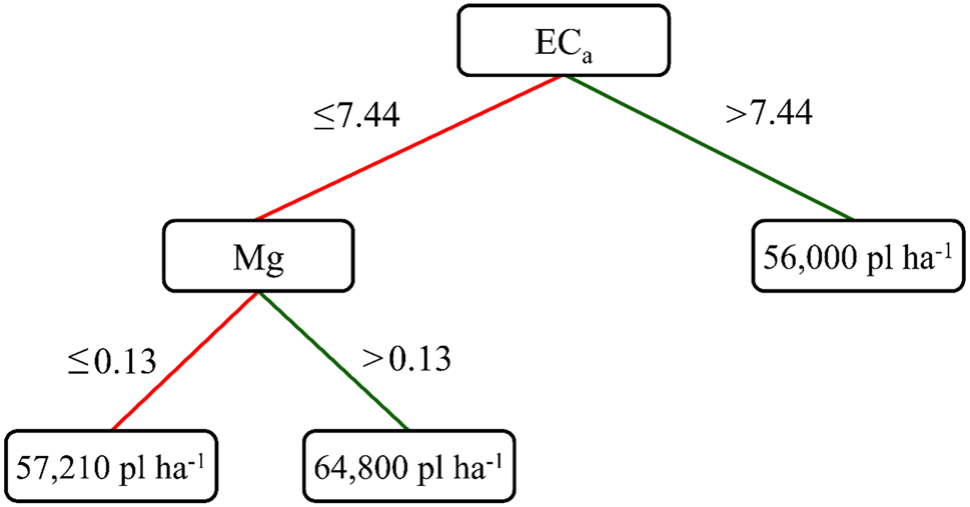
Decision tree generated using apparent electrical conductivity (EC_a_) and magnesium (Mg) as the variables selected by the trial analysis. The software used to create this figure was Genes (v1999.2019.44, http://arquivo.ufv.br/dbg/genes/gdown1.htm).

The trial analysis selected the soil attributes with the highest cause-effect relationship with GY, EC_a_, and Mg. These attributes were used to generate a decision tree (Fig. 7), which yields the plant population to look for to maximize GY. At this time, the actual populations of the experimental plots ceased to be treatments and became variables to define which maize populations should be used for each condition of the soil attributes. The number of nodes used was equal to two and provided a correlation of 0.72 in the training stage (80% of the data) and 0.73 in the validation stage (20% of the data) between estimated and observed values for GY. These results provided reliable recommendations for the optimum plant population based on the soil attributes selected by the trial analysis.

Soil attributes that maximized grain yield identified by the trial analysis were used to generate the decision tree to predict the sowing rate to be used. The first step was to observe the EC_a_ value to determine the population that would maximize GY. For EC_a_ values greater than 7.44 mS m^−1^, the ideal population size would be 56,000 plants ha^−1^, while for EC_a_ values lower than 7.44 mS m^−1^, the Mg levels would determine the ideal population size; specifically, for Mg levels greater and lower than 0.13 g kg^−1^, the ideal population sizes would be 64,800 and 57,210 plants ha^−1^, respectively.

Given the increasing demand for food and energy, monitoring and predicting grain yield is essential for food security. The prediction of maize yield is of great interest for market behavior, government policy, and increased global food security. Therefore, the development of techniques that allow accurate and timely monitoring of crop growth and early prediction of yield is crucial for crop management to materialize such high yields. An outstanding technique for monitoring crop growth and predicting crop yield is remote sensing, which provides cost-effective, timely, and accurate information on crop status.

### VRS validation

The experiment was validated at sites C and D, totaling 103 ha in 2019. VRS (Fig. 2) was performed based on the results obtained from the plots of the 2018 agricultural year in site C. Meanwhile, site D (94.5 ha) was sown in a CVS (Fig. 2) compared to VRS performed at site C.

**Figure 8:**
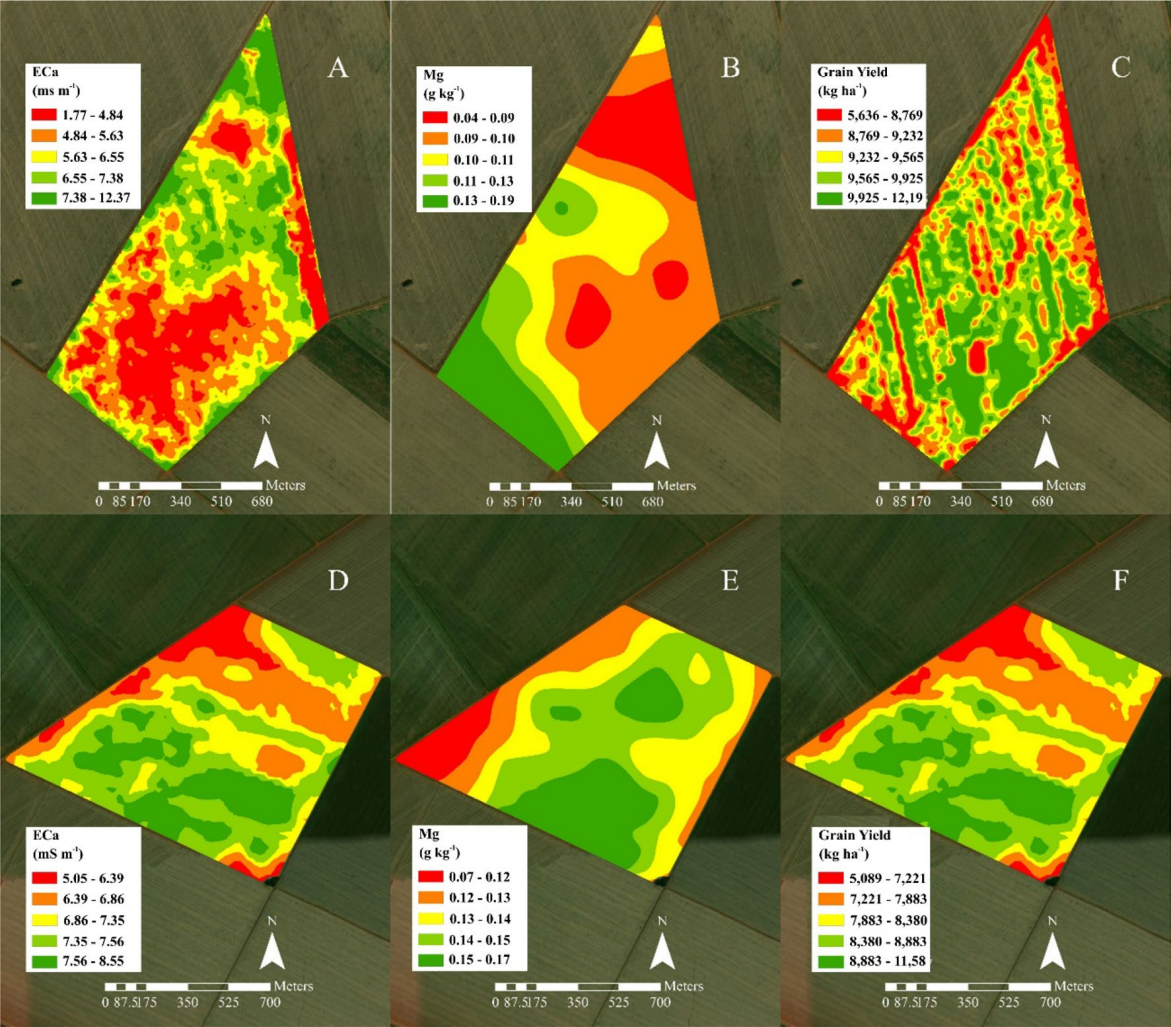
Maps of EC_a_ (**A**, **D**) and Mg (**B**, **E**) used in the decision tree and grain yield (GY, **C**, **F**) for sites **B** and **C** using VRS, respectively. The software used to create this figure was ArcGIS Desktop (v10.5, https://desktop.arcgis.com/en/system-requirements/10.5/).

The contrast analysis was performed between VRS and CVS to verify the accuracy of the decision tree. The four treatments were the three populations predicted in the decision tree (Fig. 8) and fixed-rate conventional sowing. Analysis of variance was performed, and the contrast between VRS and CVS was estimated (Table 2).

**Table 2:** Summary of analysis of variance results for maize grain yield obtained in a validation plot with variable rate seeding *versus* conventional seeding (recommended population).

Variation source	Degrees of freedom	Medium square	Means (kg ha^−1^)
Sites	4	23,167,919.18*	–
VRS	3	657,605.63^ns^	9,723.47
Contrast VRS *x* CVS	1	102,698,859.84*	8,290.30
Residue	195	686,072.42	–
Coefficient of variation	–	9.19	–

There was no statistical difference between the plant populations predicted by the decision tree (VRS). That is, the less fertile soil had a yield similar to that of the most fertile soil. However, the VRS and CVS contrast was significant, as VRS showed the highest mean than CVS. The decision tree effectively predicted the population that would maximize grain yield over the conventional rate. When using conventional fixed population seeding, grain yield would be reduced due to specific tree attributes (EC_a_ and Mg).

Figure 8 shows the decision tree attribute maps and productivity of areas 2 and 3.

The relative deviation coefficient (RDC) (Table 3) expresses the dissimilarity between two maps in the module^24^. All variable values were converted to percentage values to compare the different units. The grain yield map was considered as the reference (standard) for comparison with the other maps.

**Table 3:** Similarities between maps by relative deviation coefficient (RDC) in relation to grain yield (GY), soil apparent electrical-conductivity (EC_a_), and soil magnesium (Mg) content for variable rate seeding (upper diagonal) *versus* conventional seeding (CVS—lower diagonal).

Variable	GY	EC_a_	Mg
GY	–	21.19	8.96
EC_a_	16.53	–	10.31
Mg	11.24	12.69	–

The results in Table 3 prove the assertiveness of the trial analysis (Fig. 6), where the Mg map was more similar to the GY map in both areas. Figure 7 also demonstrates this, but the data that comprise the Pearson correlation network did not consider the spatial distribution of the data. Several authors have confirmed the assertiveness of RDC to measure the similarity between georeferenced maps^24,30,31^.

## Conclusions

CVI and GNDVI vegetation indices were the most highly correlated VIs with corn grain yield.

The generated decision tree unequivocally determined the plant populations that maximized maize grain yield using soil magnesium and electric conductivity levels under the local experimental conditions.
